# Molecular architecture and function of the hemidesmosome

**DOI:** 10.1007/s00441-014-2061-z

**Published:** 2014-12-09

**Authors:** Gernot Walko, Maria J. Castañón, Gerhard Wiche

**Affiliations:** 1 Centre for Stem Cells and Regenerative Medicine, King’s College London School of Medicine, 28th Floor, Tower Wing, Guy’s Hospital, Great Maze Pond London, SE1 9RT UK; 2 Department of Biochemistry and Cell Biology, Max F. Perutz Laboratories, University of Vienna, Dr. Bohr-Gasse 9, 1030 Vienna, Austria

**Keywords:** Hemidesmosomes, Plectin, Integrin, Bullous pemphigoid antigen, Epidermolysis bullosa

## Abstract

Hemidesmosomes are multiprotein complexes that facilitate the stable adhesion of basal epithelial cells to the underlying basement membrane. The mechanical stability of hemidesmosomes relies on multiple interactions of a few protein components that form a membrane-embedded tightly-ordered complex. The core of this complex is provided by integrin α6β4 and P1a, an isoform of the cytoskeletal linker protein plectin that is specifically associated with hemidesmosomes. Integrin α6β4 binds to the extracellular matrix protein laminin-332, whereas P1a forms a bridge to the cytoplasmic keratin intermediate filament network. Other important components are BPAG1e, the epithelial isoform of bullous pemphigoid antigen 1, BPAG2, a collagen-type transmembrane protein and CD151. Inherited or acquired diseases in which essential components of the hemidesmosome are missing or structurally altered result in tissue fragility and blistering. Modulation of hemidesmosome function is of crucial importance for a variety of biological processes, such as terminal differentiation of basal keratinocytes and keratinocyte migration during wound healing and carcinoma invasion. Here, we review the molecular characteristics of the proteins that make up the hemidesmosome core structure and summarize the current knowledge about how their assembly and turnover are regulated by transcriptional and post-translational mechanisms.

## Introduction

Hemidesmosomes (HDs) are highly specialized integrin-mediated epithelial attachment structures that make cells firmly adhere to the extracellular matrix by establishing a link between the underlying basement membrane (BM) and the internal mechanical stress-resilient keratin intermediate filament (IF) network. Although HDs are defined by their ultrastructural appearance, two types of HDs, type I and II, can be distinguished on the basis of their protein components (Hieda et al. 1992). Classical type I HDs, found in stratified and pseudostratified epithelia, for example in the epidermis, consist in five major components, namely integrin α6β4, plectin isoform 1a (P1a), tetraspanin CD151, bullous pemphigoid antigen (BPAG)1 isoform e (BPAG1e, also called BP230) and BPAG2 (also called BP180 or type XVII collagen) (Owaribe et al. 1990). Type II HDs, found in simple epithelia such as that of the intestine, consist of integrin α6β4 and plectin and lack the two BP antigens (Fontao et al. 1999; Uematsu et al. 1994).

**Fig. 1:**
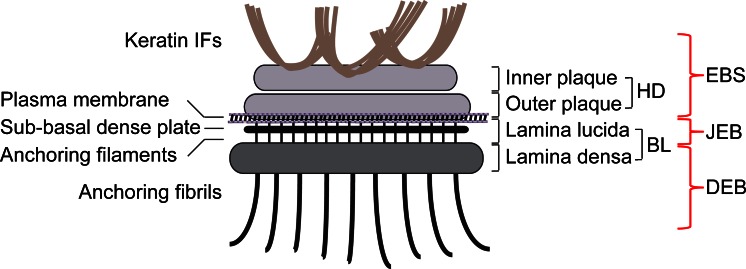
Schematic representation of a hemidesmosome and location of blisters within the skin in different types of EB. Keratin IFs are attached to the inner plaque of the hemidesmosome (HD). The outer plaque is parallel to the inner plaque, is slightly larger and lies on the plasma membrane of basal keratinocytes. Adjacent to the membrane are an electron-clear zone, the lamina lucida and an electron-dense zone, the lamina densa, which together make up the basal lamina (BL). Within the lamina lucida and directly below the plasma membrane, there is a thin electron dense line corresponding to the sub-basal dense plate. Fine anchoring filaments originate at HDs, traverse the lamina lucida and insert into the lamina densa. From the lamina densa collagenous anchoring fibrils extend into the dermal tissue. Outer brackets on the right indicate blister formation at different levels within the skin as is characteristic for the three major types of EB: EBS, within the epidermis; JEB, at the dermal-epidermal junction within the lamina lucida; DEB, in the upper layers of the dermis

Stable attachment of basal epidermal keratinocytes to the BM through HDs is of fundamental importance for maintaining skin integrity and epidermal homeostasis. Inherited or acquired diseases in which any of the HD components are affected lead to a variety of skin blistering disorders, collectively known as epidermolysis bullosa (EB) and characterized by tissue separation with blister formation within different layers of the skin. Based on where the tissue separation occurs, EB has been divided into three main categories: EB simplex (EBS), junctional EB (JEB) and dystrophic EB (DEB). In EBS, tissue separation occurs within the epidermis, in JEB, within the lamina lucida (between the dermis and epidermis) and in DEB, in the papillary dermis at the level of the anchoring fibrils (Fig. 1) (Jonkman 1999; Bruckner-Tuderman 2010; Fine et al. 2014). Mutations in at least 12 distinct genes encoding structural components of HDs have been identified as a causative of these different types of EB (Fine et al. 2014). Common clinical features include extreme skin fragility and the development of blisters and erosions in response to slight mechanical trauma. Additional extracutaneous complications associated with different variants of EB include corneal erosions, tracheal erosion and gastrointestinal blisters, dental abnormalities, nail dystrophy, esophageal strictures, pyloric atresia, muscular dystrophy and musculoskeletal deformities especially in the mittens (Pulkkinen and Uitto 1999; Fine and Mellerio 2009a, b). EBS is the most common type of EB, ranging in severity from very severe to relatively mild; most cases are caused by dominant mutations in the keratin genes *KRT5* or *KRT14*, followed by recessive mutations in the genes coding for plectin (*PLEC*) and BPAG1e (*DYS*). JEB is caused by mutations in the integrin α6 (*ITGA6*), integrin β4 (*ITGB4*), BPAG2 (*COL17A1*) and laminin 322 (*LAMA3*, *LAMB3*, *LAMC2*) genes; JEB is a severe blistering disease with recessive inheritance. DEB is caused by mutations in the collagen VII gene (*COL7A1*); the recessive inherited form is the most severe type of EB. While intact HDs are required to connect the epidermis to the underlying dermis, remodeling and dissolution of HDs are important for a variety of biological processes, such as during terminal differentiation when basal keratinocytes detach from the BM to allow for their migration toward the suprabasal epidermal layers and for migration of keratinocytes during wound healing and carcinoma invasion of skin. In these processes, cells disassemble their HDs in order to loosen their tight attachment to the basement membrane and become migratory (Wilhelmsen et al. 2006; Margadant et al. 2010; Hopkinson et al. 2014). In contrast to skin, keratinocytes and other epithelial cells in culture fail to assemble bona fide HDs. Rather, HD-enriched protein complexes (HPCs), which have also been termed stable anchoring complexes (SACs), are found along the substrate-attached basal surface of such cells (Carter et al. 1990; Ozawa et al. 2010). HPCs are not static, exhibiting dynamic properties during cell migration (Geuijen and Sonnenberg 2002; Tsuruta et al. 2003; Ozawa et al. 2010).

In this review, we will summarize our current knowledge about the molecular properties and functions of the individual protein components that make up the HD core structure. Further, we will focus on what is known about the mechanisms of assembly, stabilization and turnover of HDs, with special attention attributed to post-translational and transcriptional regulation.

## Cellular architecture of hemidesmosomes

Ultrastructural examination of intact skin reveals HDs dispersed all along the BM zone displaying a characteristic tripartite structure with inner and outer electron-dense plaques separated by a less dense zone (Tidman and Eady 1984, 1986; Kelly 1966) (Fig. 1). IFs assembled from basal cell keratins K5 and K14 associate with the inner plaque of HDs via binding to P1a and BPAG1e (Rezniczek et al. 1998; Litjens et al. 2006; Walko et al. 2011). P1a and BPAG1e form critical links with transmembrane protein complexes that form the outer HD plaque and comprise integrin α6β4, BPAG2 and CD151 (Fig. 1). Immediately beneath the basal keratinocyte plasma membrane lies an electron-lucent zone, the lamina lucida and an electron-dense layer comprising a closely packed fibrous network called the lamina densa (Briggaman and Wheeler 1975). Below the HD and parallel to the basal plasma membrane, a thin electron-dense line termed the sub-basal dense plate can be observed (Tidman and Eady 1984, 1986); it is optimally visualized in skin samples subjected to high-pressure cryoimmobilization (Reipert et al. 2004). Traversing the lamina lucida zone, subjacent to HDs, are thin anchoring filaments that extend into the lamina densa (Fig. 1). Laminin-332 is found at the border between the upper lamina densa of HDs and the lower lamina lucida at the base of anchoring filaments, which also comprises BPAG2 (Masunaga et al. 1996, 1997). Beneath the lamina densa, most of the collagen VII molecules form semi-circular loop structures, known as anchoring fibrils, that originate and terminate in the lamina densa (Shimizu et al. 1997; McMillan et al. 2003; Breitkreutz et al. 2013). In the dermis, lamina densa-anchored fibrils link or encircle dermal collagen fibers or other components, providing tight anchorage of the basal lamina in the underlying structures.

## Molecular architecture of the hemidesmosome–basement membrane connection

### Integrin α6β4 and laminin-332

**Fig. 2:**
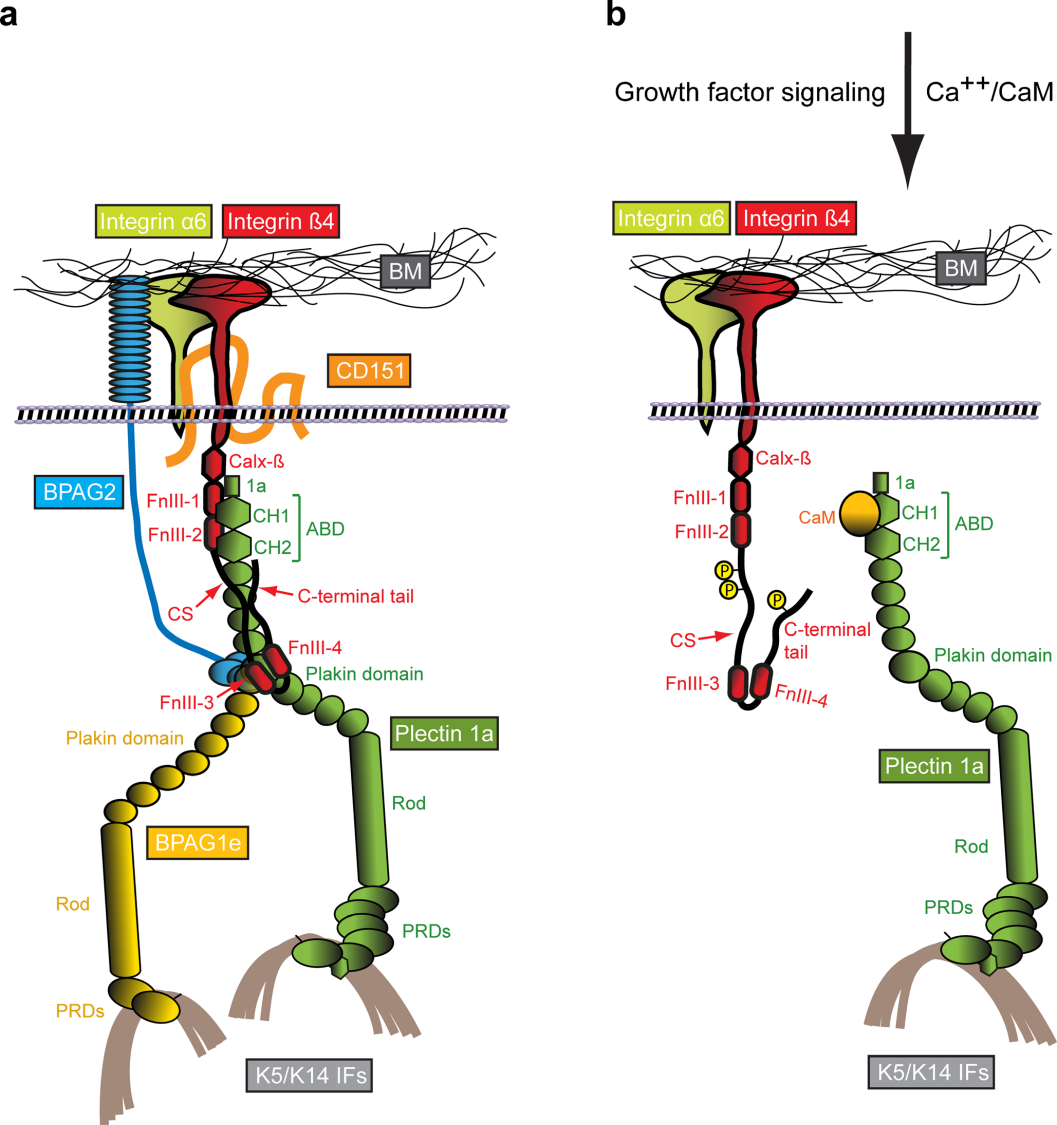
Molecular organization of HDs and a model of the HD disassembly mechanism. **a** Schematically drawn are the six key components of type I HDs (integrin subunits α6 and β4, CD151, BPAG2, BPAG1e and plectin 1a), the extracellular basement membrane (BM) containing the ligand laminin-332 and the intracellular binding partner, intermediate filaments (IFs) of the keratin (K5/K14) type. The various protein domains involved in binding of integrin β4 to plectin 1a and in the linkage of the HD complex to keratin IFs are indicated. **b** Mechanism of growth factor-induced dissociation of the integrin β4–plectin 1a complex. For simplicity, only integrin α6β4, plectin 1a and K5/K14 IFs are shown. Growth factor-induced phosphorylation of serine residues in the C-terminal integrin β4 tail and CS domains results in the dissociation of integrin β4 from plectin 1a’s plakin and ABD domains. Subsequent interaction of plectin 1a’s ABD with the Ca^++^-bound form of calmodulin (CaM) prevents re-association of the integrin β4-plectin 1a complex. 1a, isoform-specific N-terminal domain preceding the ABD of plectin 1a; Calx-β, Na^+^–Ca^2+^ exchanger motif; CH1,2, calponin homology domains 1 and 2; CS, connecting segment; FnIII-1,-2,-3,-4, fibronectin type III domains 1–4; PRDs, plectin repeat domains

Integrin α6β4 is one of the two transmembrane components of HDs (Fig. 2a). It acts as a laminin-332 receptor. Like other members of the integrin family of extracellular matrix (ECM) receptors, integrin α6β4 is a non-covalent heterodimer formed by two type I (C terminus is intracellular) transmembrane subunits, α and β (Sonnenberg et al. 1991). The genes encoding the α6 and β4 subunits have been mapped to chromosomes 2q31.1 and 17q25.1, respectively (Hogervorst et al. 1991; Iacovacci et al. 1997). Integrin α6β4 is widely expressed in epithelia where it is found in HDs but it is also expressed in several non-epithelial cell types, including endothelial cells, astrocytes, neurons and Schwann cells (Wagner et al. 1997; Nodari et al. 2008; Su et al. 2008; Van der Zee et al. 2008; Welser-Alves et al. 2013). Integrin α6 is a conventional integrin α subunit, whereas integrin β4 has a unique cytoplasmic domain that is much larger (>1,000 residues) than that of other integrin β subunits and shares no similarity with them (Hogervorst et al. 1990; Tamura et al. 1990). The cytoplasmic domain of integrin β4 is composed of five globular domains: a membrane-proximal Na^+^–Ca^2+^ (Calx-β) exchanger motif and two pairs of fibronectin type III (FnIII) domains (FnIII-1,2 and FnIII-3,4); the two pairs of FnIII domains are separated by a region named the connecting segment (CS) and a C-terminal tail extends downstream of FnIII-4 (Hogervorst et al. 1990; Suzuki and Naitoh 1990). The cytoplasmic domain of integrin β4 mediates most of the intracellular interactions of the receptor, including those with the plakin family members P1a and BPAG1e, as well as with the transmembrane protein BPAG2 (Fig. 2a). Via binding to P1a and BPAG1e, integrin α6β4 associates with the keratin IF cytoskeleton rather than with actin filaments, making this integrin heterodimer unique among the other family members (Rezniczek et al. 1998; Fontao et al. 2001; Koster et al. 2004). The α6 subunit consists in a long N-terminal extracellular domain followed by the transmembrane domain and a short intracellular domain (Hogervorst et al. 1991). The extracellular domain of integrin α6 harbors binding sites for BP180, CD151 and laminin-322 (Hopkinson et al. 1995, 1998; Kazarov et al. 2002). The large cytoplasmic domain of integrin β4 also interacts with a number of signaling intermediates involved in the regulation of cell proliferation and survival (Margadant et al. 2010). However, whether integrin α6β4 is capable of signaling when it resides within the HD has not been clarified and it seems more likely that it plays such a role when not incorporated into the HD structure, such as during keratinocyte migration or carcinoma cell invasion (Wilhelmsen et al. 2006).

The absence of integrin α6β4 in genetically modified mice results in loss of HDs and hence of stable epidermal adhesion (Dowling et al. 1996; Georges-Labouesse et al. 1996; van der Neut et al. 1996; Raymond et al. 2005; Niculescu et al. 2011). The same is true for humans that suffer from junctional epidermolysis bullosa with pyloric atresia (JEB-PA) caused by mutations in either the integrin α6 or β4 subunit. Such mutations can result in devastating diseases characterized by severe skin fragility and blistering (Chung and Uitto 2010a; Fine et al. 2014).

The extracellular moiety of integrin α6β4 binds to laminins (Fig. 2a), having a preference for the epithelial-basement membrane-specific variant laminin-332 (formerly known as kalinin or laminin 5) (Niessen et al. 1994; Rousselle and Aumailley 1994; Spinardi et al. 1995). In the skin, laminin-332 is the major component of anchoring filaments that function as a supramolecular bridge between basal keratinocytes of the epidermis and the underlying dermis. Laminin-332 consists in three disulfide-linked polypeptide chains (α3, β3, γ2) that assemble within the endoplasmic reticulum into a cross-shaped heterotrimer (Aumailley 2013). The genes encoding the laminin-332 subunits, *LAMA3, LAMB3* and *LAMC2*, are located on human chromosomes 18q11.2, 1q32.2 and 1q25.3, respectively. Laminin-332 is synthesized by keratinocytes as a high molecular weight precursor protein of 460 kDa (Marinkovich et al. 1992). After secretion and deposition into the ECM, laminin-332 undergoes proteolytic cleavage of domains located within the α3 and γ2 chains. These maturation events are essential for the supramolecular integration of laminin-332 into the BM (Rousselle and Beck 2013). Each laminin subunit has several functional domains that mediate their interaction with other ECM molecules and cell-surface receptors. The globular domain at the C terminus of the α3 subunit binds to integrin α6β4, linking the basal surface of basal keratinocytes with the dermal–epidermal BM, while the globular domain of the β3 subunit binds to collagen VII in the anchoring fibrils. These interactions are stabilized intracellularly via the association of integrin α6β4 and BPAG2 with keratin IFs through P1a and BPAG1e (Fig. 2a) and of collagen VII-containing anchoring fibrils (Rousselle et al. 1997; Rousselle and Beck 2013) to dermal collagen fibrils, on the dermal site, thus securing the adhesion of the dermal-epidermal BM to the dermal extracellular matrix (Brittingham et al. 2006; Wegener et al. 2013). Mutations in the genes encoding the laminin subunits severely affect the structure of HDs and result in generalized JEB. The absence of any of the subunits prevents heterotrimer assembly and leads to complete absence of laminin-332 in the BM. The consequences are diminished dermal–epidermal adhesion at the level of the BM and mechanically induced skin blistering and fragility in both humans and animal models (Ryan et al. 1999; Meng et al. 2003; Bruckner-Tuderman et al. 2010; Kiritsi et al. 2013; Fine et al. 2014).

### BPAG2 and CD151

BPAG2 is the second transmembrane component of HDs. This protein was first identified as an antigen that reacted with autoantibodies of patients afflicted with the disease bullous pemphigoid (BP) and several years later its role in inherited generalized intermediate JEB was uncovered (Diaz et al. 1990; Li et al. 1993; Has and Kern 2010). The gene encoding BPAG2, *COL17A*, has been mapped to the long arm of chromosome 10q24.3 (Li et al. 1991, 1993). Expression of BPAG2 is restricted to stratified, pseudostratified and transitional epithelia (Fairley et al. 1995). BPAG2 is a homotrimeric transmembrane protein of type II (intracellular N terminus). Each molecule consists in three 180-kD collagen alpha-1(XVII) chains that are characterized by a globular intracellular domain, a short transmembrane stretch and an extracellular C-terminal domain composed of 15 collagen repeats separated by 16 noncollagenous (NC) subdomains. The membrane proximal domain NC16A serves as a nucleus for the formations of a flexible collagen-like triple helix (Hirako et al. 1996). BPAG2’s intracellular domain lies within the outer plaque of the HD and the extracellular domain spans the lamina lucida with the tail residing in the lamina densa. The extracellular domain of BPAG2 is constitutively shed from the cell surface by action of ADAM proteases, resulting in the formation of a ∼120-kD protein fragment (often referred to as ectodomain or LAD-1), which becomes incorporated into the BM (Franzke et al. 2002). The functional consequences of BPAG2 processing are still not fully understood; the current theory is that it may be important for regulation of keratinocyte detachment from the basement membrane (Hirako et al. 2003; Nishie et al. 2011). BPAG2 has been shown to contain multiple binding sites for HD proteins, including plectin, BPAG1e and integrin β4 in its cytoplasmic domain and integrin α6 and laminin-332 in the extracellular domain (Fig. 2a) (Hopkinson et al. 1995, 1998; Borradori et al. 1997; Schaapveld et al. 1998; Koster et al. 2003). Although the binding of BPAG2 to laminin-332 has been shown in vitro, this interaction by itself is not sufficiently strong to mediate adhesion of cells to laminin-332 in the absence of integrin α6β4 (Van den Bergh et al. 2011). Mutations in the *COL17A* gene are associated with diminished epidermal adhesion and with skin blistering. The disease type is JEB, including several subtypes (Chung and Uitto 2010b; Has and Kern 2010; Kiritsi et al. 2011; Fine et al. 2014). Ultrastructural abnormalities include rudimentary HDs and the separation of basal keratinocytes from the underlying basement membrane. The human disease is phenocopied by knockout of the *COL17A* gene in mice (Nishie et al. 2007).

CD151 is a cell surface protein that belongs to the tetraspan superfamily of transmembrane proteins. These proteins are involved in cell adhesion, migration and signaling (Zoller 2009). All tetraspanin proteins share a similar structure characterized by four transmembrane domains forming a small and a large extracellular loop, with short intracellular N- and C-terminal tails (Maecker et al. 1997). The human *CD151* gene is located on chromosome 11p15.5 and is expressed in the basal keratinocytes of skin and other epithelia and in the vascular endothelium (Hasegawa et al. 1997; Sincock et al. 1997). In keratinocytes, CD151 but no other tetraspanins, colocalize with HDs (Sterk et al. 2000). CD151 interacts with integrin α6 via its large extracellular loop (Fig. 2a) and, at least ex vivo, appears to be involved in HD formation and turnover (Sterk et al. 2000). Mutations in the CD151 gene are associated with nephropathy and skin fragility in humans (Karamatic Crew et al. 2004). In contrast to humans, knockout of CD151 in mice has no apparent effect on HD formation and stability, although wound healing is impaired (Wright et al. 2004; Cowin et al. 2006). Absence of CD151 in cultured keratinocytes was shown to stabilize HPCs by interfering with protein kinase C (PKC)α-mediated disassembly (Li et al. 2013). Consequently, CD151 was found to play a key role in skin squamous cell carcinoma (Li et al. 2013; Winterwood et al. 2006).

## The hemidesmosome–intermediate filament cytoskeleton connection

### Plectin isoform 1a (P1a)

On their cytoplasmic face, HDs are linked to the keratin cytoskeleton via two members of the plakin family of cytoskeletal linker proteins. One of them, the 500-kDa protein plectin, is expressed in a wide variety of tissues and cell types, where it orchestrates the networking, interactions and dynamics of various types of IFs, thereby crucially affecting their functionality (Wiche and Winter 2011; Castañón et al. 2013). Encoded by single genes on chromosomes 8q24 and 15 in humans and mice, respectively (Liu et al. 1996; Fuchs et al. 1999), plectin molecules exhibit a multidomain structure that enables them to interact with a vast array of different proteins. Plectin binding partners comprise components of cellular junctions (desmosomes, HDs, tight junctions, focal adhesions, neuromuscular junctions, costameres), the plasma, nuclear and mitochondrial membranes, the cytoskeleton (myofibers, IFs, microtubules, cytolinkers), centrosomes, the proteasome/apoptosis machinery and signaling pathways (Castañón et al. 2013). Electron microscopy of single molecules (Foisner and Wiche 1987) and structure predictions, based on the amino acid sequence deduced from plectin cDNA, revealed a tripartite structure with a central 200-nm-long coiled-coil rod domain flanked by globular N- and C-terminal domains (Wiche et al. 1991). The rod exhibits a regular 10.5 periodicity in acidic and basic residues that are out of phase by 180 degrees, which improves rod function and promotes self-association (Green et al. 1992). The N-terminal domain comprises an ABD, the C-terminal domain 6 plectin repeat domains (PRDs) (Janda et al. 2001). Most of plectin’s interaction sites reside within its N- and C-terminal globular domains, including a multifunctional actin-binding domain (ABD) and a universal IF-binding site at opposite ends. Due to alternative splicing of its gene transcripts, plectin is expressed in the form of an unusual number of isoforms with differing N-terminal head domains. These variable parts dictate the differential subcellular targeting of the isoforms. Through their ability to recruit (via their common C-terminal domains) IFs of different types, plectin molecules provide strategically located IF anchorage sites within the cytoplasm of cells (Wiche and Winter 2011).

Plectin was identified as a prominent component of HDs and HPCs in epithelial tissues and cell lines early on by immunofluorescence and immunoelectron microscopy of frozen tissue sections (Wiche et al. 1983, 1984) as well as biochemical studies (Hieda et al. 1992; Skalli et al. 1994) (regarding protein identities, see Herrmann and Wiche 1987; Okumura et al. 1999; Clubb et al. 2000). The essential role of plectin for formation and homeostasis of HDs became clearly evident when patients suffering from EBS-MD, a disease characterized by severe skin blistering (due to rupture and lysis of basal cell layer keratinocytes) and late onset muscular dystrophy were shown to carry mutations in the plectin gene (Rezniczek et al. 2010; Winter and Wiche 2013). Skin biopsies of such patients revealed that the inner plaque structure of HDs was missing or structurally compromised (Gache et al. 1996; Smith et al. 1996). Similarly, the targeted inactivation of the plectin gene in mice led to a severe skin blistering phenotype, characterized by a drastic reduction in the number of HDs, clear signs of cell rupture occurring at the level of the inner plate structure and reduced keratin filament association with inner plate structures preserved in non-ruptured areas of skin (Andrä et al. 1997; Ackerl et al. 2007). First evidence for the association of a specific isoform of plectin (P1a) with HDs was provided by immunogold electron microscopy of rat skin applying domain-specific and isoform-specific antibodies (Rezniczek et al. 1998). The selective association of P1a with HDs was clearly established when it was shown that (1) P1a transcripts account for ∼70 % of all plectin transcripts detected in cultured mouse keratinocytes, (2) in contrast to other isoforms, P1a shows colocalization with HDs and HPCs in skin epidermis and cultured keratinocytes and (3) HD defects in plectin-deficient cells could be rescued by forced expression of P1a but not of other isoforms (Andrä et al. 2003; Rezniczek et al. 2003; Walko et al. 2011). Consistent with P1a’s dominant role in maintaining HD integrity, mice deficient in P1c (which accounts for only ∼20 % of all plectin transcripts expressed in basal keratinocytes but is the most abundant isoform when considering the whole epidermis) show no skin blistering phenotype (Fuchs et al. 2009). Aside from its presumed role in HD-targeting, the N-terminal isoform-specific sequence enables P1a to bind to CaM in an isoform-specific and Ca^2+^-dependent manner, a mechanism that promotes HD disassembly during Ca^2+^-induced keratinocyte differentiation (Kostan et al. 2009; see Fig. 2b).

Plectin has been shown to directly bind to the cytoplasmic tail domain of integrin β4 via multiple sites although the primary contact takes place between the ABD of plectin and the first pair of FNIII domains of integrin β4 (Rezniczek et al. 1998; Geerts et al. 1999; Koster et al. 2004) (Fig. 2a). The crystal structures of plectin’s ABD as well as that of the primary plectin–integrin β4 complex have been resolved (Garcia-Alvarez et al. 2003; Sevcik et al. 2004; de Pereda et al. 2009a). These analyses showed that the ABD of plectin consists in two calponin homology (CH) domains arranged in a closed conformation and that plectin binds to integrin β4 through its CH1 domain, whereas integrin β4 binds to plectin through its FNIII-2 domain (de Pereda et al. 2009a). Two missense mutations in the FNIII-2 domain of integrin β4, R1225H and R1282W, that abolish plectin–integrin β4 interaction result in EBS with pyloric atresia (Koster et al. 2001, 2003; de Pereda et al. 2009b). Plectin directly interacts with keratins K5/K14 via its universal IF-binding site located in its C-terminal domain in the linker region between PRDs 5 and 6 (Nikolic et al. 1996; Steinböck et al. 2000) (Fig. 2a). A recent study suggests that additional C-terminal domains contribute to this association acting synergistically to strengthen K5/K14 binding (Bouameur et al. 2014b).

The most revealing insights into the role of P1a, in formation, homeostasis and stabilization of HDs, were gained by studying the dominant skin blistering disease EBS-Ogna (Koss-Harnes et al. 2002). In contrast to autosomal recessive mutations in plectin, which account for the multisystem disorders epidermolysis bullosa simplex (EBS) associated with muscular dystrophy (EBS-MD), pyloric atresia (EBS-PA) and congenital myasthenia (EBS-CMS), the rare EBS-Ogna mutation manifests exclusively in skin, where it leads to blister formation in basal cells immediately above HDs (Koss-Harnes et al. 2002). The mutation is a heterozygous C.T transition leading to a pR2000W substitution in plectin’s rod domain, which mediates the dimerization of the protein via coiled-coil formation. In studies on the pathomechanism of EBS-Ogna, it has recently been shown that this amino acid substitution exposes residues sensitive to intracellular proteolysis, resulting in impaired HD formation. The specificity of the proteolytic HD-associated-P1a cleavage is brought about by the spatio-temporal activation of calpain and serine proteases in the epidermis but not, e.g., in muscle tissue (Walko et al. 2011). The mechanism proposed implies that the mutated residue in Ogna-plectin locally alters the conformation of plectin’s dimeric rod domain by unfolding its coiled-coil structure around the site of the mutation, rendering plectin more susceptible to proteolytic degradation by basal cell membrane-associated keratinocyte proteases. The reduced level of HD-recruited P1a becomes then insufficient to promote formation of HDs in adequate numbers and of optimal stability for keratin IF network anchorage, eventually leading to skin fragility in response to mechanical stress. Recently, patients diagnosed with a similarly exclusive skin (EBS) phenotype as that of Ogna patients were shown to be carriers of mutations in exon 1a, which encodes the isoform-specific sequence of P1a (Jonkman and Lemmink 2012).

**Fig. 3:**
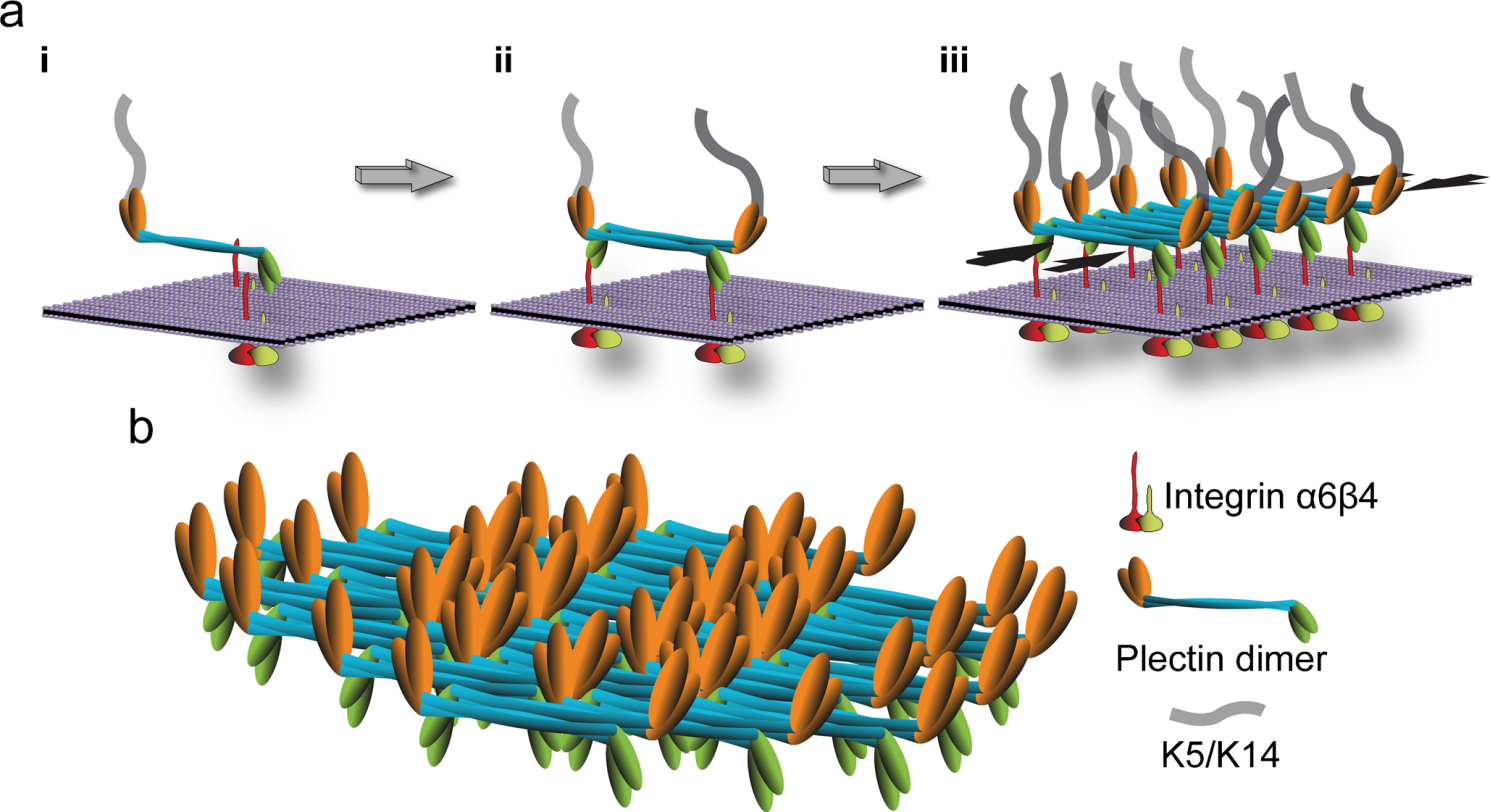
Hypothetical model of HD stabilization through plectin self-association. **a** Three consecutive stages (*i*–*iii*) of inner plaque formation are depicted; for simplicity, only integrin α6β4 and plectin 1a molecules are shown. In a first step (*i*) a parallel plectin 1a dimer binds, via its N-terminal β4-binding domains (*green*), to the cytoplasmic tail (*red*) of plasma membrane (*violet*)-embedded integrin β4; plectin’s C-terminal domain (*orange*) makes the connection to keratin filaments. In a second step (*ii*), two plectin 1a dimers form a tetramer by lateral association of their central rod domains in an anti-parallel fashion, creating integrin β4 and K5/K14 binding sites at both ends of the tetramer. Further self-association of plectin rod segments leads to oligomeric sheet-like structures (*iii*). Note that self-association of plectin 1a molecules could lead to the clustering of integrin α6β4 (as depicted), or vice versa, clustered integrins may facilitate the focal recruitment of plectin molecules promoting their alignment and lateral association; also, targeting of plectin 1a molecules to integrin β4-could occur in their keratin IF-bound (as indicated) or unbound state.* Opposing black arrows*, plaque-stabilizing force component provided by laterally associated rod segments. **b** Structural model of an inner plaque assembled by the staggered lateral association of plectin dimers. Note the focal density of potential K5/K14 and integrin β4 binding sites on opposite sides of the plate structure. This model is based on the ultrastructural analysis of paracrystalline structures formed from recombinant full-length versions of plectin’s rod domain and the lateral association potential of intact plectin molecules isolated from cells (Walko et al. 2011)

Beyond shedding light on the pathomechanism, studies on the Ogna mutation revealed a previously unknown feature of plectin molecules that potentially has great impact on HD structure and stability. It was reported that the central α-helical rod domains of coiled-coil plectin dimers can further associate laterally into extraordinary stable (paracrystalline) polymers. Based on these findings focal self-association of plectin molecules was proposed as a mechanism contributing to HD stabilization. According to the proposed model (Fig. 3), the recruitment of P1a to laminin-clustered integrin α6β4 molecules in developing HDs could lead to the proximal alignment of plectin’s rod domains, favoring their interaction and the assembly of compact highly ordered polymeric plectin structures. Thus, integrin α6β4-induced oligomerization of dimeric plectin molecules by lateral association of their RDs within the inner plaque structure would literally add another dimension (horizontal) to plectin’s HD-stabilizing potential. In this scenario, sufficient numbers of stable keratin-IF-linked HDs would only form if enough intact full-length P1a molecules are available for oligomerization. Whether other cytolinker proteins, such as the HD component BPAG1e, are also capable of lateral self-association and whether plectin can form hetero-oligomers with such proteins remains to be shown. Hetero-oligomer formation has previously been reported for plakins such as periplakin and envoplakin (Kalinin et al. 2004) and preliminary experiments using recombinant rod domains of plectin and BPAG1e indicate heterodimerization (authors’ unpublished results). Interestingly, a potential for self-interaction was also reported for integrin β4’s cytoplasmic domains (Rezniczek et al. 1998), a feature that may promote the clustering and alignment of integrins, providing a force component for HD stabilization similar to that of laterally-associated plectin rods.

### Bullous pemphigoid antigen 1 isoform e (BPAG1e)

The second plakin family member that links HD integrins to the K5/K14 cytoskeleton is BPAG1e, an epidermally expressed isoform of BPAG1. BPAG1 was originally identified as an antigen with immunoreactivity to autoantibodies of patients suffering from bullous pemphigoid, an autoimmune sub-epidermal skin-blistering disease (Stanley et al. 1988). The mouse ortholog of BPAG1 is also called dystonin in consideration of the fact that mutations in its gene cause sensory neuron degeneration in the mouse mutant dystonia musculorum (dt) (Brown et al. 1995). The BPAG1 genes (*DST*) in humans and mice are localized on chromosomes 6p12.1 and 1, respectively (Sawamura et al. 1990; Copeland et al. 1993). Similar to plectin, multiple exons in the BPAG1 gene are the basis of several protein isoforms generated by alternative splicing of transcripts. Four major variants, BPAG1a, BPAG1b, BPAG1e and BPAG1n, have been identified, which are expressed in the nervous system (BPAG1a and BPAG1n), muscle (BPAG1b) and epidermis (BPAG1e). They vary considerably in size and structure with additional variability provided by alternative first coding exons for at least some of them (Suozzi et al. 2012). BPAG1e, the only isoform associated with HD, is a 230-kD protein with an overall structure very similar to that of plectin. It contains a 140-nm-long α-helical coiled-coil rod domain (that shares with plectin its 10.5 periodicities of negatively and positively charged amino acid residues) flanked by an N-terminal plakin domain and a C-terminal region comprising two (compared to plectin’s six) PRDs (Green et al. 1992; Tang et al. 1996). However, in contrast to plectin, BPAG1 has no ABD (Suozzi et al. 2012).

BPAG1e has been shown to bind to the HD components integrin α6β4, BPAG2 and keratins K5 and K14 (Bouameur et al. 2014a), thereby further stabilizing the HD junction. These interactions have been mapped to the N-terminal region of BPAG1e and the CS and second pair of FNIII repeats of integrin β4 (Koster et al. 2003), the N-terminal domains of BPAG1e and BPAG2 (Hopkinson and Jones 2000) and the C-terminal domain of BPAG1e (K5/K14) (Guo et al. 1995; Fontao et al. 2003) (Fig. 2a). Additionally, the rod domain of BPAG1e can form heterodimers with its plectin counterpart in vitro (unpublished results).

Two recent reports describe the first mutations in the *DST* gene causing a new form of autosomal recessive EBS, classified as EBS-2 (Groves et al. 2010; Liu et al. 2012). The mutations, p.Q1124X and R1249X, in the rod domain of BPAG1a, lead to the loss of HD inner plaques, unattached keratin filaments and a complete absence of skin immunostaining for BPAG1-e, as well as reduced labeling for plectin, integrin β4 and BPAG2. The affected individuals had a lifelong history of trauma-induced spontaneous blisters and erosions, particularly around the ankles, feet, hands and elbows (Groves et al. 2010; Liu et al. 2012). The knockout of the DST/BPAG1 gene in mice had shown a similar skin phenotype, as HDs lacked inner plate structures and had no keratins attached (Guo et al. 1995).

## Protein–protein interactions leading to the assembly and stabilization of hemidesmosomes

Several observations support the notion that the binding of integrin α6β4 to laminin-332 is a crucial initial step in HD assembly. First, patients who suffer from JEB-PA or generalized intermediate/severe JEB due to mutations in their genes for either integrin α6 or integrin β4, or laminin-332, show dermo-epidermal separation and only rudimentary HDs can be found in their skin (Shinkuma et al. 2011). Second, detachment of the epidermis from the underlying dermis is also observed in knockout mice with deficiencies in either integrin α6 or integrin β4; and in these cases not even rudimentary HDs can be observed (Dowling et al. 1996; Georges-Labouesse et al. 1996; van der Neut et al. 1996). Third, the compromised HPC formation found in keratinocyte cultures derived from JEB-PA and generalized severe JEB patients can be rescued by re-expression of the intact proteins (Gagnoux-Palacios et al. 1996, 1997; Melo et al. 2014; Schaapveld et al. 1998).

The next step in HD assembly after the initial integrin α6β4–laminin-332 binding is the interaction of P1a with integrin β4 via P1a’s ABD and the integrin’s first pair of FnIII repeats and part of its CS (Geerts et al. 1999; Koster et al. 2001, 2004). The importance of this interaction is highlighted by the reduced number and hypoplastic nature of HDs found in the skin of JEB-PA patients with R1281W or R1225H mutations in the integrin β4 FnIII-2 repeat and by the compromised formation of HPCs in keratinocytes expressing such mutants (de Pereda et al. 2009b; Koster et al. 2001, 2003). Likewise, in skin of plectin-null mice as well as of EBS-MD and EBS-Ogna patients with P1a deficiencies, HDs were found to be hypoplastic and numerically reduced (Gache et al. 1996; Andrä et al. 1997; McMillan et al. 1998; Koss-Harnes et al. 2002). The specific targeting mechanism of P1a, compared, e.g., to P1c, remains to be solved. A higher binding affinity of P1a’s ABD to integrin β4 compared to actin (Kostan et al. 2009) at least explains why P1a in keratinocytes shows preferential binding to the integrin. The interaction between P1a’s ABD and integrin β4 is strengthened by the additional interaction of plectin’s plakin domain (common to all plectin isoforms) with sites located in the C-terminal domain of the CS and within the C-terminal FnIII-4 repeat of integrin β4 ( Rezniczek et al. 1998; Koster et al. 2004). In the tertiary structure of integrin β4, these additional plectin-binding sites are in close proximity to each other and mediate an intramolecular association of integrin β4 (Rezniczek et al. 1998; Schaapveld et al. 1998), thereby positioning the integrin β4 cytoplasmic domain for an optimal interaction with the plakin domain (Frijns et al. 2012).

BPAG2 has been suggested to bind intracellularly to plectin’s plakin domain and to the FnIII-3 repeat of integrin β4 via different binding sites located in its cytoplasmic domain, once a stable P1a–integrin β4 complex has been formed (Borradori et al. 1997; Schaapveld et al. 1998; Koster et al. 2003). Whereas the extracellular domain of BPAG2 was shown to bind to laminin-332 (Tasanen et al. 2004; Nishie et al. 2011; Van den Bergh et al. 2011), it is dispensable for BPAG2’s recruitment to HDs (Hopkinson et al. 1995; Borradori et al. 1997). The unusual localization of BPAG2 in the apical region of keratinocytes, derived from a plectin-deficient EBS-MD patient (Gache et al. 1996), demonstrated that BPAG2 is efficiently recruited to the HD only when plectin is present. Likewise, less P1a was found in integrin β4-positive HPCs of keratinocytes derived from a BPAG2-deficient generalized severe JEB patient (Koster et al. 2003). However, the interaction between BPAG2 and P1a evidently is not sufficiently strong to induce the formation of HPCs in the absence of integrin α6β4, because in integrin β4-deficient JEB-PA cells, HPC formation could not be observed, despite BPAG2 and plectin being expressed normally in these cells (Niessen et al. 1996; Schaapveld et al. 1998). In contrast to the in vitro situation represented by cultured keratinocytes, rudimentary HDs can still be found in the skin of patients deficient in plectin (Gache et al. 1996; Koss-Harnes et al. 2002) or integrin α6β4 (Niessen et al. 1996). Possible explanations for this apparent in vitro/in vivo discrepancy could be that in vivo BPAG2 interacts either with the integrin α6 subunit or with an unknown ligand located in the BM (Koster et al. 2003; Wilhelmsen et al. 2006). Collectively, these findings support the notion that the BPAG2–plectin interaction, though not required for induction of HDs formation, strengthens the ternary integrin α6β4–plectin-BPAG2 complex and acts as a platform for the incorporation of BPAG1e (Koster et al. 2003).

The next recruit to HPCs was found to be BPAG1e that associates via its plakin domain with BPAG2 and integrin β4; the integrin β4 domain involved in this interaction comprises the C-terminal 21 amino acids of the CS and the second pair of FnIII repeats (Schaapveld et al. 1998; Hopkinson and Jones 2000; Koster et al. 2003). In addition to the multiple associations exerted by the cytoplasmic domain of the integrin β4 subunit, the extracellular domain of the α6 subunit also interacts with BPAG2 and CD151 (Hopkinson et al. 1995, 1998; Sterk et al. 2000).

The K5/K14 IF network linkage provided by the C-terminal domains of both P1a and BPAG1e is crucial for the ability of HDs to withstand mechanical stress, as trauma-induced rupture of basal epidermal keratinocytes in skin of humans and mice with P1a or BPAG1e deficiencies occurs where keratin IFs insert into the HD inner plaque structure (Guo et al. 1995; Gache et al. 1996; Ändra et al. 1997; Koss-Harnes et al. 2002; Groves et al. 2010; Liu et al. 2012). Moreover, in cultured keratinocytes, the absence of either P1a or keratin IFs was shown to compromise HPC formation (Osmanagic-Myers et al. 2006; Walko et al. 2011; Seltmann et al. 2013), suggesting that for the stabilization of HDs their anchorage to the keratin IF network is also of importance. The IF-binding domain of BPAG1e binds specifically to K5/K14 (Fontao et al. 2003), whereas the universal IF-binding site of P1a shows stronger binding to other types of IFs, such as vimentin filaments (Steinböck et al. 2000). However, as recently shown, plectin’s binding strength to K5/K14 is increased by additional C-terminal keratin-binding sites that act synergistically (Bouameur et al. 2014b). The K5/K14-binding specificity of BAPG1e and the presence of type II HD-related structures lacking BPAG1e and BPAG2 in simple epithelial tissues (Fontao et al. 1999) suggest that the incorporation of both BPAG proteins into skin type I HDs occurred to meet the greater mechanical stress protection requirements of tissues such as skin. Whereas the P1a/BAPG1e-mediated anchorage of the ternary integrin α6β4/P1a/BPAG2 complex to the K5/K14 IF network could provide a HD-stabilizing force component, which is more or less perpendicular to the plasma membrane, the lateral association of multiple P1a dimers, combined with the presumptive P1a-BPAG1e hetero-oligomerization and the self-association of integrin β4 molecules (see above) could generate additional force components along the plasma membrane (Fig. 3) (Walko et al. 2011).

## Regulation of hemidesmosome turnover by post-translational mechanisms

HD components are not expressed in suprabasal keratinocytes of the epidermis, indicating that keratinocytes in the proliferative basal cell compartment dissolve their HDs once they commit to terminal differentiation and stratify out into the suprabasal cell layers (Fuchs and Raghavan 2002). Disassembly of HDs also occurs transiently when keratinocytes become migratory in order to repopulate a skin wound or during carcinoma invasion (Janes and Watt 2006; Wilhelmsen et al. 2006; Hopkinson et al. 2014). Transient disassembly is achieved by post-translational mechanisms including phosphorylation-dependent disruption of protein–protein interactions, whereas permanent dissolution of HDs during terminal differentiation also involves repression of gene transcription (see next section) and proteolytic cleavage of HD components.

Several growth factors have been implicated in regulating transient HD disassembly in response to wound healing, such as epidermal growth factor (EGF), hepatocyte growth factor and macrophage-stimulating protein (MSP) (Santoro et al. 2003; Lipscomb and Mercurio 2005; Litjens et al. 2006; Margadant et al. 2008). In cultured keratinocytes, these factors collectively stimulate phosphorylation of integrin β4 on serine and threonine residues in the CS and C-terminal tail domains, leading to HPC disassembly (Rabinovitz et al. 2004; Wilhelmsen et al. 2007; Germain et al. 2009; Frijns et al. 2010, 2012; Kashyap et al. 2011). A current model of EGF-induced HPC disassembly suggests that phosphorylation of integrin β4 on T1736 by PKD1 or other CAMK-like kinases results in the dissociation of the C-terminal tail of integrin β4 and the plakin domain of P1a, whereas phosphorylation of integrin β4 at S1356 and S1364 by ERK1/2 and p90RSK1/2 kinases, respectively, disrupts the interaction between the first pair of integrin β4’s FNIII repeats and P1a’s ABD (Frijns et al. 2012). The serine/threonine protein kinases effecting the dissolution of HPCs are counterbalanced by serine/threonine protein phosphatases, including calcineurin (Kashyap and Rabinovitz 2012). Whereas in cultured keratinocytes, S1356, S1364 and T1736 are not significantly phosphorylated until the cells are stimulated with EGF, a different site (S1424, phosphorylated by PKCα) in the CS domain, shows a high level of constitutive phosphorylation in non-stimulated keratinocytes (Germain et al. 2009) and thus might be responsible for the dynamic nature of HPCs (Geuijen and Sonnenberg 2002; Germain et al. 2009). Dissolution of HPCs during terminal differentiation could also be linked to phosphorylation of integrin β4 by PKCδ (Alt et al. 2001; Adhikary et al. 2010). For complete disassociation of the integrin β4–P1a complex to occur, in addition to phosphorylation of integrin β4, calcium-controlled binding of calmodulin to P1a’s ABD might play an important role (Kostan et al. 2009). Although HPC and HD stability seems to be mainly dependent on the interactions of integrin β4 with P1a, additional associations must be broken for full complex dissolution, including those of integrin β4 with BPAG1e and BPAG2. The phosphorylation of BPAG2 by PKC, leading to its translocation from HPCs, as well as the phosphorylation of integrin α6 by PKC have been reported (Alt et al. 2001; Kitajima et al. 1992, 1999). In studies on HD dissolution, EGF has been on center stage but it should be noted that in cultured keratinocytes EGF alone does not induce complete disruption of HPCs. It is likely that, under physiological conditions, such as during wound healing of skin, additional growth factors are involved in inducing the activity of protein kinases that contribute to HD disassembly. A likely candidate would be MSP, a ligand for the receptor tyrosine kinase MST1R (macrophage-stimulating 1 receptor, also known as RON), which regulates multiple processes in keratinocytes, including proliferation, survival and migration. Keratinocyte stimulation with MSP results in serine phosphorylation of integrin α6β4, causing its 14-3-3 protein-dependent mobilization to lamellipodia and the partial breakdown of HPCs (Santoro et al. 2003). In pancreatic cells, MSP-induced RON signaling leads to disruption of plectin–integrin α6β4 interaction, HPC disassembly and enhanced cell migration (Yu et al. 2012).

Several reports have suggested that integrin β4 is also phosphorylated on tyrosine residues. Although in most of these studies carcinoma cells overexpressing the EGF receptor were used (Mariotti et al. 2001; Trusolino et al. 2001; Merdek et al. 2007), evidence has been presented that tyrosine phosphorylation may also occur in normal keratinocytes (Mainiero et al. 1995, 1996). Considering that in carcinoma cells integrin β4 is not primarily localized at the basal side of the cell but instead is distributed over the entire plasma membrane (contrary to keratinocytes), kinases that normally do not have access to it might effect its aberrant phosphorylation. This could explain its assumed function as an adapter protein for tyrosine kinases and their associated signaling proteins in cancer cells (Lipscomb and Mercurio 2005; Wilhelmsen et al. 2006).

During calcium-induced terminal differentiation of cultured keratinocytes, integrin α6β4 was found to be rapidly downregulated by the combined action of phosphorylation-induced protein dissociation, proteolytic processing and decreased gene transcription, leading to the disappearance of HPCs within a few hours (Tennenbaum et al. 1996; Kostan et al. 2009). The protein level of P1a also decreased during differentiation albeit at a slower rate compared to integrin β4; this slower decay may have been due to P1a’s continued association with keratins (Kostan et al. 2009). Proteolytic processing of both, integrin β4 and P1a, during terminal differentiation was shown to be mediated by the calcium-activated protease calpain and there is evidence that the same mechanism also operates in vivo (Giancotti et al. 1992; Potts et al. 1994; Walko et al. 2011). Proteolytic processing of integrin β4 and P1a is likely to serve in preventing re-association of the proteins and HPC formation during keratinocyte differentiation.

## Control of hemidesmosome dissolution by repression of gene expression.

Regulation of integrin α6β4 expression in proliferating versus differentiating keratinocytes is mostly transcriptionally and to some extent also epigenetically, controlled. Whereas high expression levels of p63 in basal keratinocytes contribute to maintaining the expression of integrin β4 but not of integrin α6 (Carroll et al. 2006; Mulder et al. 2012), induction of canonical Notch signaling during commitment of basal keratinocytes to terminal differentiation represses (via RBP-J) the transcription of both integrin subunits (Blanpain et al. 2006; Restivo et al. 2011). Consequently, as a result of reduced HD levels, focal epidermal detachment was observed in mice with hyperactivated Notch signaling in basal keratinocytes (Ambler and Watt 2010). Although the integrin α6 gene is not targeted by p63, its expression in keratinocytes was shown to be under epigenetic control by the MORF histone acetyl-transferase complex (Mulder et al. 2012). Repression of integrin α6β4 gene transcription and a resulting reduction of HDs in skin were also identified as part of a cellular response to increased expression of c-Myc in basal keratinocytes (Frye et al. 2003; Gebhardt et al. 2006). Finally, there is also evidence that SoxF transcription factors play a role in regulating the expression of integrin α6β4 as well as plectin during development (Oommen et al. 2012). However, the downregulation of P1a during terminal differentiation of cultured keratinocytes was found not to involve transcriptional regulation (Kostan et al. 2009).

## In conclusion

Since the first protein constituents of HDs were isolated and characterized some 25 years ago, tremendous progress has been made in understanding the structure–function relationship of hemidesmosomal proteins, how they assemble into HDs and how they mediate adhesion to the ECM and the underlying skin layer. Considerable progress has also been achieved in elucidating the mechanisms of HD disassembly during cell proliferation, wound healing and carcinoma invasion. The molecular cloning of the hemidesmosomal proteins has been instrumental in identifying the etiology of skin blistering diseases and provided the basis for their diagnosis. Also of great value was the generation of animal models that mimic the various EB diseases; they have helped to dissect the mechanisms underlying the diseases and they will be invaluable in developing therapeutic strategies. Challenging tasks for future research will include the 3-D structural analysis of purified protein complexes as well as in situ HDs by innovative electron microscopy such as cryoelectron microscopy and cryotomography of sectioned basal keratinocytes. Such analyses will deepen our understanding of the molecular mechanisms leading to epithelial cell attachment and epithelial tissues reinforcement and will allow the evaluation of models for HD stabilization that are based on the lateral association of HD components as reviewed in this article. Other challenges will be the full elucidation of signaling pathways that effect the crosstalk between HDs and other epithelial cellular junctions (particularly focal adhesions) in keratinocyte migration during carcinoma invasion. For mechanistic studies of this kind, it would be of advantage to generate and use 3D-culture models (multi-cellular spheroids) that more closely resemble the in vivo environment than the more conventional monolayer cultures. Finally, EBS patients will benefit from whole exome sequencing for the rapid identification of the causative genes and of rare structural and functional genetic variants leading to EB.
